# Chronic Kidney Disease-Mineral and Bone Disorder

**DOI:** 10.1155/2017/4875042

**Published:** 2017-06-19

**Authors:** Wen-Chin Lee, Xiong-Zhong Ruan, You-Ying Chau, Jin-Bor Chen

**Affiliations:** ^1^Division of Nephrology, Department of Internal Medicine, Kaohsiung Chang Gung Memorial Hospital and Chang Gung University College of Medicine, Kaohsiung, Taiwan; ^2^Centre for Nephrology & Urology, Department of Physiology, Shenzhen University Health Science Center, Shenzhen, China; ^3^Centre for Nephrology, University College London, London, UK; ^4^Centre for Cardiovascular Science, Queen's Medical Research Institute, Edinburgh, UK

Chronic kidney disease-mineral and bone disorder (CKD-MBD) is common in CKD patients and plays pivotal roles in the altered bone metabolism and vascular/valvular calcification. Several guidelines on the management of CKD-MBD have been published but large gaps between guidelines and clinical practice still exist. The field is swamped with information coming in the forms of basic and clinical research articles. Timely published review articles and focused special issues on this topic provide quick and valuable tools for the busy medical professionals looking after CKD patients.

In this special issue, we offer a nexus for the most updated collection of reviews and clinical studies on CKD-MBD. This issue includes three review articles and six clinical studies. For better understanding the mechanisms of CKD-MBD, Dr. Y. Iwasaki et al. provide an intensive review on molecular abnormalities underlying bone fragility in chronic kidney disease. Dr. Y.-C. Hou et al. review roles of vitamin D in uremic vascular calcification. From practical viewpoints, Dr. N.-C. Chen et al. review strategies to prevent and regress the vascular calcification in dialysis patients.

Clinical studies presented in this special issue collectively cover a broad spectrum of CKD-MBD. Dr. W.-H. Wang et al. investigate the association between parathyroid hormone, 25 (OH) vitamin D, and chronic kidney disease. Dr. Y.-P. Sun et al. analyze clinical epidemiology of mineral bone disorder markers in prevalent hemodialysis patients in Xinjiang and call for an urgent need to improve dialysis adequacy in the largest province in China. Dr. Y. Liu et al. examine the practical use of the KDIGO recommended target range of CKD-MBD markers in the real world and find that achievement of the suggested serum phosphorus level is the most important factor for lowering mortality in hemodialysis patients. In line with Dr. Y. Liu's findings, by looking at coronary artery calcification in peritoneal dialysis patients, Dr. D. Shang et al. address hyperphosphatemia and hs-CRP important initiators of vascular calcification in CKD-MBD. Parathyroidectomy (PTX) is indicated in CKD stages 3–5D with severe hyperparathyroidism that fail to respond to medical therapy. However, the impacts of PTX on serum fibroblast growth factor 23 (FGF23) and soluble Klotho levels and the optimal post-PTX intact parathyroid hormone (iPTH) levels are seldom investigated. By analyzing a larger patient cohort, Dr. S.-C. Liao et al. find no changes in serum concentrations of FGF23 and soluble Klotho in hemodialysis patients after total PTX. Dr. Q. P. Xi et al. look into the impact of post-PTX iPTH levels on all-cause mortality in dialysis patients.

Articles presented in this special issue offer novel insights into the pathogenesis of CKD-MBD and inspire new ideas in clinical management on this complex problem.

*Wen-Chin Lee* *Wen-Chin Lee* *Xiong-Zhong Ruan* *Xiong-Zhong Ruan* *You-Ying Chau* *You-Ying Chau* *Jin-Bor Chen* *Jin-Bor Chen*

